# Cell dynamics and gene expression control in tissue homeostasis and development

**DOI:** 10.15252/msb.20145549

**Published:** 2015-02-25

**Authors:** Pau Rué, Alfonso Martinez Arias

**Affiliations:** Department of Genetics, University of CambridgeCambridge, UK

**Keywords:** development, differentiation, homeostasis, stochastic cell fate, transition state

## Abstract

During tissue and organ development and maintenance, the dynamic regulation of cellular proliferation and differentiation allows cells to build highly elaborate structures. The development of the vertebrate retina or the maintenance of adult intestinal crypts, for instance, involves the arrangement of newly created cells with different phenotypes, the proportions of which need to be tightly controlled. While some of the basic principles underlying these processes developing and maintaining these organs are known, much remains to be learnt from how cells encode the necessary information and use it to attain those complex but reproducible arrangements. Here, we review the current knowledge on the principles underlying cell population dynamics during tissue development and homeostasis. In particular, we discuss how stochastic fate assignment, cell division, feedback control and cellular transition states interact during organ and tissue development and maintenance in multicellular organisms. We propose a framework, involving the existence of a transition state in which cells are more susceptible to signals that can affect their gene expression state and influence their cell fate decisions. This framework, which also applies to systems much more amenable to quantitative analysis like differentiating embryonic stem cells, links gene expression programmes with cell population dynamics.

## Introduction

The total number of cells of an organism is a tightly regulated variable. For instance, a human being contains more than 10^13^ cells (Bianconi *et al*, 2013), while a male *Caenorhabditis elegans* nematode worm is composed of exactly 1,031 cells (Sulston, 1976; Sulston & Horvitz, 1977). These cells are organized in tissues and organs with two important properties. First, their final size is well defined, emerges during development and is species specific denoting the existence of an ‘internally driven final state’ (Garcia-Bellido & de Celis, 1992; García-Bellido, 2009), which represents a growth target during development and therefore requires precisely organized proliferation of cells. Second, although most tissues are subject to a continuous loss of cellular mass due to wear and tear (Spalding *et al*, 2005), their size is maintained approximately constant for the lifetime of the organism and, in many cases, can be restored in response to severe injury. This second property suggests the existence of a self-regulated steady state or ‘homeostasis’ that keeps the number of cells constant through a balance of cell loss and proliferation. In both cases, it is not only the total number of cells that is accounted for but also their identities which, in many instances, need to be balanced for the correct function of tissues or organs. While knowing the genes associated with these events is important, it is the link between the genes and the population dynamics that will allow elucidating the molecular mechanisms that underlie these systems.

Here, we review the current knowledge on the dynamics of cell populations during homeostasis, highlight recent findings on universal patterns associated with this process and explore whether and how can these be extrapolated to developing tissues. We conclude that a key element of cell populations in homeostasis and development is the regulation of the dynamics of gene expression during the transition between different fates. This regulation takes place at the level of single cells and acts on what we call the ‘transition state’. This state provides the substrate to link population cell dynamics and gene expression.

## Cell division and differentiation: basic mechanisms and principles

**Figure 1:**
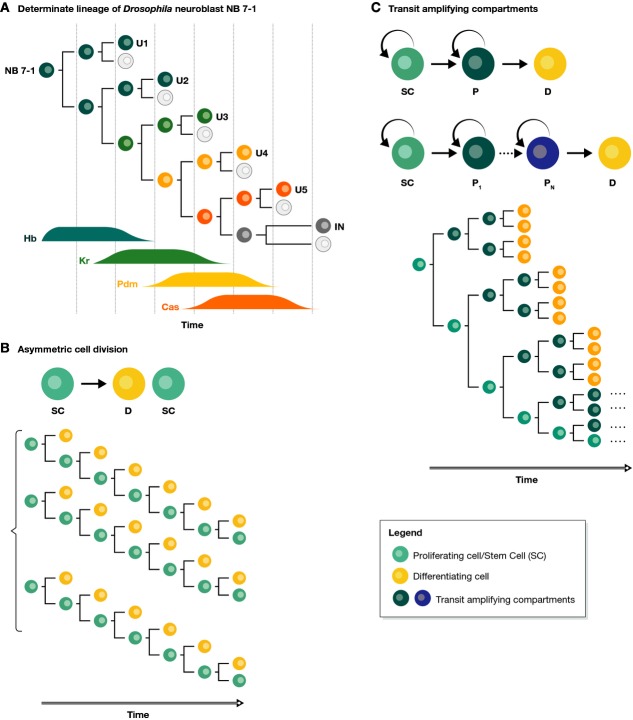
Cell proliferation and differentiation (A) The generation of neuroblast lineages in *Drosophila* is an example of a determinate process consisting of reproducible sequences of asymmetric cell divisions with changing fates. The succession of different fates upon each asymmetric cell division is controlled by a precise genetic programme on the progenitor, relying on the sequential expression of Hunchback (Hb), Kruppel (Kr), Pdm and Cas (adapted from Kohwi & Doe, 2013). (B) Asymmetric cell division is an invariant mechanism of generating differentiated progeny from stem cells where one daughter cell differentiates (D, yellow) and the other remains a stem cell (SC, teal). In homeostatic lineages, invariant asymmetry leads to homogeneous cell lineages. (C) Transit-amplifying cells are progenitors derived from stem cells that retain a proliferative capacity for a few division rounds before differentiating. SC: stem cell; D: differentiated cell; P, P_1_,…,P_N_: progenitors.

There are three ways to coordinate cell division and cell identity/fate. The first one is exemplified by eutelic organisms like *C. elegans*, in which every division is associated with the generation of two different cells and this process is iterated over time (Sulston, 1976; Sulston & Horvitz, 1977). The number of divisions is exquisitely regulated, such that each tissue is the result of a defined lineage built from a sequence of asymmetric cell divisions, that is, each gives rise to two different cells, and underpinned by a hardwired genetic programme (Gönczy, 2008; Knoblich, 2008). This strategy can also be found in other systems like the embryonic central neural system (CNS) of insects (Kohwi & Doe, 2013) where each neuroblast sequentially divides asymmetrically to self-renew and generates a differentiating ganglion mother cell, which can further divide and generate two differentiated neural cells (Fig1A). The whole process is associated with a gene expression programme running on each neuroblast, which involves the sequential expression of Hunchback, Kruppel, Pdm and Cas. These highly deterministic systems are usually associated with small and fast-developing embryos and have little regulative capacity: when a cell is lost, it is not replenished. At the other extreme, there are situations in which a group of cells make copies of themselves over a period of time and are given specific identities as the tissue grows by virtue of global cues, for example the imaginal discs of *Drosophila* (Wartlick *et al*, 2011). In these cases, there is no recognizable pattern relating cellular proliferation and fate assignment. In between these two extremes, there is a collection of dynamic behaviours exemplified by systems of growth driven by stem/progenitor cells, which divide asymmetrically to generate a cell that remains undifferentiated and thus sustains a naive state, and another cell that differentiates (Fig1B). In some instances, in tissues with wear and tear, these same cell populations maintain tissue homeostasis and have received increased attention over the last few years (Pellettieri & Sánchez Alvarado, 2007; Simons & Clevers, 2011).

The possible interactions between the dynamics of cell proliferation and differentiation allow for a rich collection of behaviours that are exploited in the biology of organs and tissues and that can be analysed using simple mathematical models (see Box 1). These models indicate that, to fulfil the requirements of developing and homeostatic systems, the rates of proliferation and differentiation need to be extremely well balanced and coupled to the events that determine cell fates.

**Box 1: Symmetric/asymmetric cell divisions and stochastic differentiation of homeostasis**

Stochastic models of stem/progenitor cell dynamics

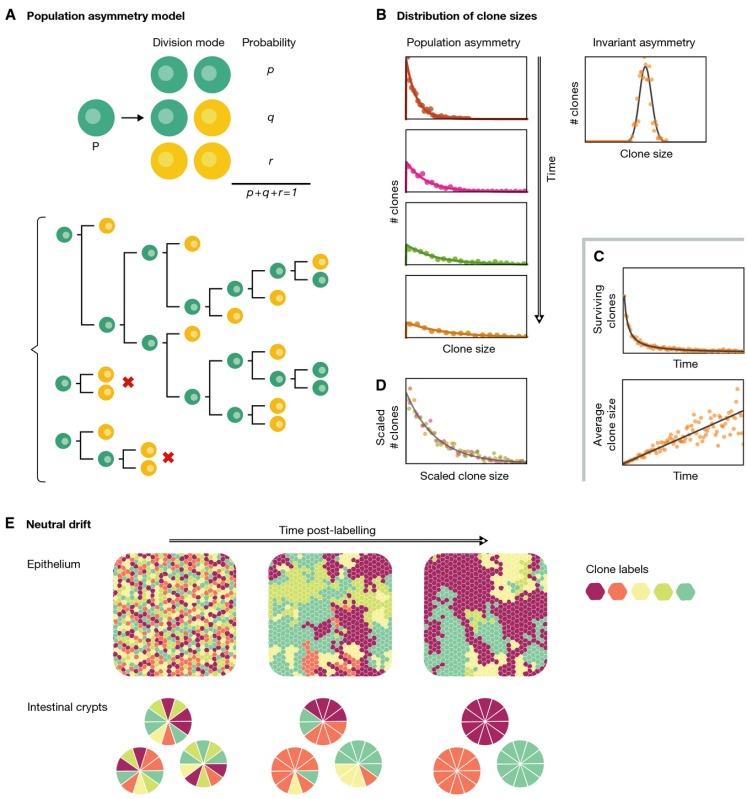

Homeostatic stem and progenitor cell populations are examples of indeterminate systems in which cells can divide and differentiate continuously. According to the fate adopted by the daughter cells, their divisions can be classified as symmetric proliferating (PP), symmetric differentiating (DD) or asymmetric (PD) (Potten & Loeffler, 1990) with a distribution of frequencies *p*,*q* and *r,* respectively (*p + q + r = *1*,* see panel A), that can be estimated experimentally. The outcome of each individual division is a priori unpredictable and thus can be deemed to be stochastic. The model that results from these considerations is known as the population asymmetry model (panel A). The dynamics of these simple rules of division and differentiation can be accounted statistically by means of branching processes as first introduced by Till, McCulloch and Siminovich (Till *et al*, 1963), whom only considered symmetric divisions, and rediscovered and further developed, later on, by Simons and colleagues (Clayton *et al*, 2007; Klein *et al*, 2007). This framework assumes that cells undergo division in any of the three possible modes outlined above. As a result, in each PP division, the number of SCs increases by one; in PD division, the number of stem cells remains unaltered, and in DD divisions, there is a net loss of one proliferative cell. From these basic premises, it follows that, in homeostatic systems, the fraction of proliferative and differentiative divisions has to be balanced, that is, *p = r*, and thus *q = *1* −* 2*r*. Any small deviation from this balance would lead, within a few cell cycles, to either a collapse or an exponential growth of the stem cell population. One extreme case of this process is that of pure asymmetric divisions (*p = r =* 0*, q = *1, Fig1A), in which the number of stem cells is predicted to be constant, and the number of DD proportional to it. Such systems are not robust, and any tissue damage would be irreversible. Alternatively, if the outcome of each division event is assumed to be random—in the sense of not being predictable—and independent of the result of previous divisions, a Markovian treatment of the branching process leads to a number of prediction in the dynamics and statistics of clonal populations of cells (reviewed in Klein & Simons, 2011). First, if the system exhibits symmetric differentiating divisions (*r > *0), there will be a continuous and unavoidable loss of clones, as stem and progenitor cells might get extinct by chance (upper panel in C and clones with a red cross in A). However, in homeostatic conditions (*p = r*), the decreasing number of surviving clones is compensated for by their continuous average growth (lower panel in C). A second prediction is that the distribution of sizes of clones of the same age is much broader than that expected from an invariant asymmetry model (panels in B), and it gets increasingly broader with time. Moreover, in the long term, the distribution exhibits scaling: if at each time one normalizes the distribution by the average clone size, the distribution becomes time invariant (bottom panel in B). These predictions have been extensively studied in used in several homeostatic systems (reviewed in Klein & Simons, 2011) and proved successful in reproducing experimental data, particularly the distribution of clone sizes and the probability of clone extinction. The stochastic cell division process leads to a scenario of neutral competition among clones, which leads to a coarsening of the surviving clones. This phenomena has been observed experimentally using clone labelling in mouse intestinal crypts, where individual clones can eventually take over a whole crypt (neutral fixation), and in epithelia, where a chimaeric pattern arises in the long term (panel E, adapted from Klein & Simons, 2011). In such cases, stochastic models correctly predict times of clone extinction and expansion (Klein & Simons, 2011).

### From single cells to populations: the structure and dynamics of adult stem cell populations

#### Stochastic structure of clonal growth in homeostatic systems

Strictly deterministic lineages cannot easily explain homeostasis in the case of tissues consisting of large and indeterminate numbers of cells that, nevertheless, maintain a defined size, such as the haematopoietic system. Every day an average human being replenishes 1% of the red blood cells in the bloodstream. This represents > 10^9^ cells, each of which is the result of a carefully controlled lineage tree that contributes to maintaining homeostasis (Bryder *et al*, 2006). Part of the answer to how this is achieved lies in the work of J. Till and E. McCulloch in the 60s (Till & McCulloch, 1961), who restored the ability of irradiated mice to make blood, by injecting bone marrow cells. In these experiments, Till and McCulloch observed in the spleen of the injected mice the appearance of colonies resulting from a founding effect of haematopoietic stem cells (SCs). The colonies exhibited a large disparity in the number of cells, and this led them to propose a stochastic *birth* and *death* model for SC activity (Till *et al*, 1963, see Box 1), namely each dividing SC would either give rise to two SCs (birth event) or differentiate and exit self-renewal (death event, see Box 1). This simple model, based on the assumption that there might be loose control of cell fate assignment during division, was able to explain the observed variability in the number of stem cells per clone. However, the model also illustrated the need for a control mechanism that regulates the probabilities of division versus differentiation at the population level: any slight divergence between proliferation and loss would lead in the short term to an exponentially growing imbalance.

Rapidly cycling ‘solid tissues’, such as skin, the intestine or the epithelium covering mucous membranes, also require a constant supply of differentiated cells (DD) in defined proportions. Analysis of these tissues (Leblond, 1981; Potten & Loeffler, 1990) led to a general model, the SC/TA model, in which a population of slow-cycling SCs maintains a population of rapidly dividing progenitor cells, the transit-amplifying (TA) compartment (Fig1C), that protects the SCs from being used up and serves as a substrate for differentiation (Potten, 1974, 1981). Further considerations in the context of the population size that these cells maintain suggested the existence of *populational asymmetry*, with some cells differentiating and others dividing symmetrically to generate two proliferative cells that could complement strictly asymmetric cell divisions at the level of single cells (Watt & Hogan, 2000).

A long-term study of the time evolution of clones in the skin of the mouse tail failed to support the well-established SC/TA compartment model and established a paradigm which is a universal feature of homeostatic systems (Clayton *et al*, 2007). Instead of confined finite-sized growth compartments, as predicted by the SC/TA model (Potten, 1974), the study revealed large variation in the number of clones and their individual size. Furthermore, the experiment revealed a continuous loss of clones that was counterbalanced by the expansion of the surviving clones, which, on average, expanded at a constant rate. A crucial observation was that the shape of the long-term distribution of clone sizes is time invariant, even though it becomes stretched according to the average size of persisting clones, that is, the clone distribution scales. Such scaling behaviour in the distribution of clone sizes is a signature of phenomena undergoing neutral drift dynamics, whereby random loss of clones due to depletion of stem cells is balanced with expanding clones (Box 2, Klein & Simons, 2011). This led to the suggestion that rather than being maintained by a population of slow-cycling stem cells, the skin of the mouse tail is maintained by a population of progenitors which divide stochastically to generate one of the three outcomes: two progenitors (PP), two DD or one of each (PD) with fixed probabilities. The stochastic structure of clonal growth is reminiscent of the notions introduced by Till and McCulloch, but the realization of the clone scaling and the calculation of the ratios of the different division types indicate the existence of a simple and reproducible process underlying the renewal of this tissue.

Similar observations have since been made in other homeostatic systems in vertebrates (Klein *et al*, 2010; Lopez-Garcia *et al*, 2010; Snippert *et al*, 2010; Kent *et al*, 2013; Kozar *et al*, 2013) as well as in *Drosophila* (De Navascués *et al*, 2012) suggesting the existence of universal patterns, that is, robust signatures shared between systems, of stem cell self-renewal (see Box 1 for details). This means that the shape of the size distribution for each of these classes is fixed by the particular mode of cellular balance and the geometry of the tissue (see Klein & Simons, 2011 for a detailed review).

**Box 2:Proliferation/differentiation control in homeostasis and development**

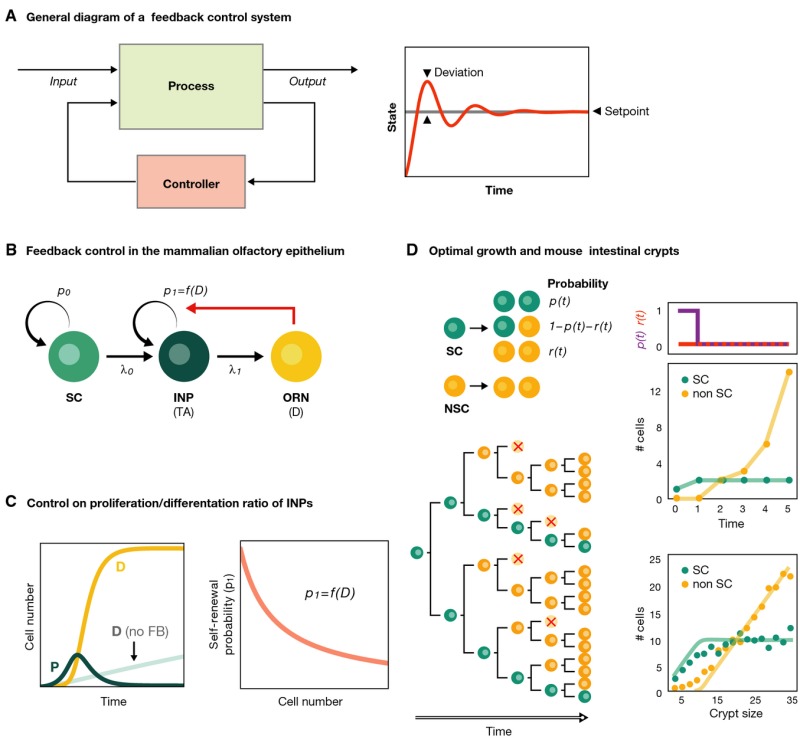

Biological systems share some features with engineered ones, in particular their tendency to aim towards and then operate within a set point, which, often, is optimal for a specific function. Homeostasis and development are examples of this behaviour and exhibit analogies with systems built to accomplish defined tasks under strict rules of robustness and optimality in performance. For instance, systems whose operation requires maintaining a particular magnitude to a fixed set point are engineered through feedback regulation whereby information on the state of the process (output) is used by a controller that feeds back to the system (input) in order to correct any deviations of the state from the set point level (panel A). If the output is off, the input will be modified to attain this set value; this guarantees the steadiness of the fixed parameter that regulates its behaviour (right plot in panel A, see Doyle *et al*, 1990). Similar mechanisms have been postulated to operate in biological systems, and it is possible to draw a useful analogy between set point and both developmental and homeostatic systems, where the principal ‘performance objectives’ are to rapidly achieve and robustly maintain a specified size or number of different cell types (Reeves & Fraser, 2009). This analogy has been used to investigate organ size control in homeostasis disruption and during development within the mammalian olfactory epithelium (OE) (Lander *et al*, 2009). This tissue undergoes constant and rapid neurogenesis by means of a well-defined linear multistage lineage in which terminally differentiated olfactory receptor neurons (ORNs) are sustained by a transit-amplifying compartment of immediate neuronal precursor (INP) cells which in turn are sustained by a stem cell (SC) compartment (panel B). The dynamics of this system can be characterized by the probabilities of self-renewal of SCs and INPs, *p*_0_ and *p*_1_, respectively, as well as their cell division rates λ_0_ and λ_1_. Analysis of quantitative data suggests that if the differentiated neurons negatively fed back onto the probabilities of proliferation/differentiation of the INPs (parameter *p*_1_ in schema B and right panel C), the steady state of the system becomes robust and it could react orders of magnitude faster to perturbations such as tissue damage [left panel C, cf. the recovery of damaged DD (solid yellow line) to the case with no feedback (pale teal line)]. Production of GDF11, a ligand of the TGFβ superfamily produced by neuronal cells and known to inhibit the production of ORNs, is capable of such feedback control (Lander *et al*, 2009). Thus, the dynamic response to punctual perturbations of an otherwise balanced system can uncover some aspects about the regulation of the homeostasis.

Optimal control theory (Donald, 1970) provides strategies towards efficiency in the achievement of specific aims and can be used to understand certain developmental systems, for example how to achieve a certain size within the shortest period of time. This question has been addressed in the context of the developing intestinal crypts of infant mice (Itzkovitz *et al*, 2012a) looking for the optimal temporal progression of the probabilities of each type of division, *p*(*t*), *q*(*t*) and *r*(*t*) (panel D, adapted from Itzkovitz *et al*, 2012a). When the SC population is not allowed to overshoot, its solution consists of two differentiated phases: one of exponential proliferation of SCs, with all divisions being symmetric, and a second one in which divisions become purely asymmetric and non-stem cells (NCSs) are generated and multiply (panel D). Any strategy other than this performs suboptimally. Remarkably, the strategy fits the data to a great extent (right bottom panel D; Itzkovitz *et al*, 2012a). This type of analyses provides deeper understanding of the cell dynamics during development and opens new question such as how to reconcile this progression towards purely asymmetric divisions during crypt maturation with the population asymmetry behaviour observed in adult mice. Further investigation with control engineering tools will surely cast some light to this problem.

#### Models of stem and progenitor cell dynamics

These observations suggest the existence of competition between equipotent progenitors, leading to a neutral drift of the clones similarly to allele drift in a population (Kimura, 1984). The models indicate that analysing the long-term behaviour of the distribution of clone numbers and sizes reveals the neutral drift (Box 1). Random drift of equipotent clones can also lead to clone fixation if the number of cells in a population is small. Monoclonality has been observed in the mammalian intestine*,* a tissue that has become a benchmark to analyse the dynamics of stem cell populations in homeostasis (Lopez-Garcia *et al*, 2010; Snippert *et al*, 2010). The continuous loss of cells from the villi on the intestine membrane is compensated by constant replenishment from a stem cell population located in crypts at the base of each villus. This population has a very well defined structure, with only a few active SCs giving rise to TA cells, which further differentiate into, among others, enterocytes and secretory cells (Lopez-Garcia *et al*, 2010; Snippert *et al*, 2010). Clone labelling of mice intestinal stem cells confirmed prior evidence that crypt SC clonality is attained in just a few weeks (Griffiths *et al*, 1988; Winton *et al*, 1988) and provided quantitative insights into the dynamics of the process. Interestingly, a simple model with a finite number of stem cells undergoing stochastic division can accurately account for the fraction of crypts that become monoclonal over time (Snippert *et al*, 2010). Continuous clonal labelling also indicates that at any given time, only a subset of stem cells in each crypt contributes to tissue homeostasis. The actively dividing stem cells per crypt turn out to be as few as five to seven, which roughly corresponds to 30–50% of the lower crypt (Kozar *et al*, 2013). Moreover, their replacement rate is kept approximately constant for at least 2 years of age, indicating that such cells do not suffer exhaustion (Kozar *et al*, 2013).

In these models, the probabilities of each class of division are either deemed to be intrinsic to the cells or locally balanced by means of cell-extrinsic mechanisms (Klein & Simons, 2011). In either case, the models indicate that the fates of the two daughter cells in each division are somehow linked: the frequencies of PP, PD and DD divisions predicted by the models are far from what would be expected if the fates of the two cells were independently assigned. These models are stochastic and, in homeostatic conditions, require an exquisite balance of the parameters controlling the cellular dynamics (i.e. are poised at criticality). Furthermore, they assume that the drivers of the system are cell intrinsic and do not address the associated biochemical mechanisms. However, the observation that, when perturbed, these experimental systems respond by changing parameters such as the fraction of PP divisions suggests that there exist control mechanisms that might go beyond those considered implicitly in these models. As discussed in the context of the olfactory epithelium in Box 2, these control systems might be central to the dynamics of the population and provide a link with the molecular and signalling mechanisms that underlie the process (Lander *et al*, 2009).

## Molecular mechanisms underlying homeostatic systems

### The existence of a ‘transition state’

Accounting for the dynamics of homeostatic populations does not explain how different fates arise, or how they are maintained and propagated. Understanding this requires a description of the molecular events associated with fate assignment in those lineages and, more specifically, with the maintenance and differentiation of stem cell populations. A first approximation to this problem is currently being pursued by isolating stem and progenitor cells from different systems and performing single-cell gene expression studies. To date, these studies have failed to identify stereotypic profiles specific to stem cells and instead have revealed broad heterogeneous distributions of gene expression often associated with the tissue to which the given stem cell contributes (e.g. Tang *et al*, 2010; Itzkovitz *et al*, 2012b; Muñoz *et al*, 2012; Pina *et al*, 2012; Guo *et al*, 2013; Kent *et al*, 2013; Tan *et al*, 2013; Yan *et al*, 2013; Turner *et al*, 2014a). In some instances, such as the mammalian intestine and the follicular and interfollicular epidermis, there seem to be different populations of stem cells with different profiles which effectively support the same function (Tan *et al*, 2013; Schepeler *et al*, 2014). The main conclusion so far is that rather than being a well-defined homogeneous profile of gene expression, the signature of a stem cell is a heterogeneous ensemble of gene expression patterns specific to the particular associated cell type. In these ensembles, cells express many differentiation genes at low and variable levels.

**Figure 2:**
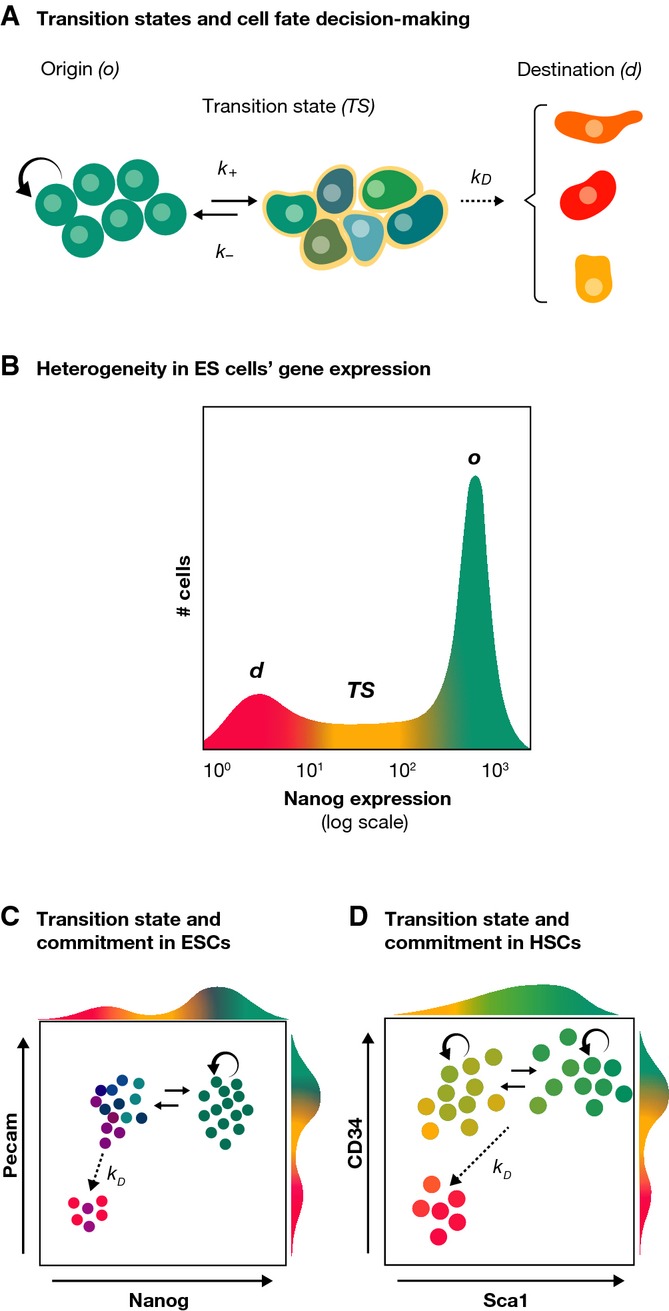
Molecular mechanisms of self-renewal and differentiation: the transition state (A) The concept of the transition state (*TS*) between an origin (*o*) and a destination (*d*) state. During a fate change, a cell goes through a TS (for details see text), which implies the existence of kinetic constants governing the transitions between different states. (B) The TS can be observed in mouse ESCs. In this case, this is shown within the framework of Nanog expression, which is heterogeneously expressed with three distinguishable populations: *o*, representing ground state pluripotency; *d*, where it is possible to find cells committed to differentiation and *TS* where cells make a choice. (C) The coexistence of committed and uncommitted cells in the Nanog:GFP d population can be revealed by looking at a second pluripotency marker, Pecam or SSEA1 in this case (Canham *et al*, 2010; Lim, 2011). (D) A similar scenario has been recently observed in blood stem cells: cells with high levels of Sca1 can self-renew and are in a state analogous to the ‘o’ state. Sca1 low cells further subdivide into two populations, which can be identified by CD34. Sca1^−^/CD34^+^ have repopulation capacity and can revert to Sca1^+^ while the Sca1^−^/CD34^−^ population consists of erythroid commited cells with no self-renewal capacity.

One interpretation of this observation is provided by the notion of multilineage priming (Hu *et al*, 1997). According to this notion, a characteristic of a stem cell population is the expression of markers of multiple lineages at low levels, which creates a landscape of differentiation potential (Moignard & Göttgens, 2014). A related view is contained in the ‘transition state’ (Fig2A), a concept derived from the observation that when cells change state during development, the decision is taken by individual cells from a ‘transition state’ (TS), in which a cell transiently exhibits a mixed identity between the states we could call origin (*o*) and destination (*d*) (Fig2A; Martinez Arias & Hayward, 2006; Muñoz Descalzo & Martinez Arias, 2012). At the TS, a cell has a probability of returning to *o* or moving to *d,* and its mixed identity is reflected in simultaneous, though variable, expression of genes from both states in the same cell. Once a cell moves from the TS towards the *d* state, the progression becomes irreversible. In a population undergoing a state transition between two states (o and d), this results in a mixture of cells in one of three states: *o*,*TS* and *d*. Such heterogeneous patterns of gene expression are often observed in developmental systems. If one associates a self-renewal rate to cells in *o* and *TS* and balances the ratios of transition of this self-renewal with differentiation, the result is something that formally resembles a stem cell population, which thus could be construed as a self-replicating transition state (Muñoz-Descalzo *et al*, 2012). The TS is a crucial step during the cell fate decision process, and this contrasts with the notion of lineage priming that merely describes a population in a steady state.

### Gene expression patterns in dynamic stem cell populations

These concepts have been illustrated and studied in mouse embryonic stem (ES) cells, clonal populations derived from mouse preimplantation blastocysts that are pluripotent and can be differentiated *in vitro* into all cell types (Smith, 2001; Nichols & Smith, 2011). ES cells can be stably propagated in culture and are characterized by heterogeneous gene expression with individual cells expressing a spectrum of genes from pluripotent to differentiation (see Fig2B and Chambers *et al*, 2007; Hayashi *et al*, 2008). It has been suggested that this heterogeneity is driven by the noisy expression of a small network of transcription factors centred on Nanog, Oct4, Sox2 and Essrb that are central to the maintenance of pluripotency (Chambers *et al*, 2007; Singh *et al*, 2007; Chambers & Tomlinson, 2009; Kalmar *et al*, 2009; Young, 2011; Abranches *et al*, 2014; Singer *et al*, 2014). The activity of this network can be read out in the distribution of Nanog expression (Kalmar *et al*, 2009; Abranches *et al*, 2014), which is characterized by three populations in dynamic equilibrium (Fig2B) with a dominance of a high Nanog pluripotent self-renewing population and a low Nanog population. While both populations contain cells that are lineage primed, only the low Nanog population includes cells that are committed to differentiation (Chambers *et al*, 2007; Kalmar *et al*, 2009; Luo *et al*, 2013; Munoz Descalzo *et al*, 2013). Remarkably, several experiments have shown that this distribution is robustly maintained and that its profile can be reconstituted even from small populations of ES cells (Chambers *et al*, 2007; Singh *et al*, 2007; Kalmar *et al*, 2009; Canham *et al*, 2010; Abranches *et al*, 2013). These states are thus dynamic and interconvertible (though the transition rates may vary with the culturing conditions), enabling cells to sample different molecular states (Kalmar *et al*, 2009; Abranches *et al*, 2013, 2014). It has been suggested that, at the molecular level, this dynamic state is fuelled by the time average of a loose connectivity of the elements of the network which creates a number of microstates [i.e. one of the many permitted states of gene expression (Garcia-Ojalvo & Martinez Arias, 2012; MacArthur & Lemischka, 2013)], some of which are compatible with self-renewal (high Nanog expression) and others with differentiation [low Nanog expression (Kalmar *et al*, 2009; Trott *et al*, 2012; Munoz Descalzo *et al*, 2013)]. The fraction of different populations is approximately constant for a given condition but changes with signalling, suggesting that it is regulated (Luo *et al*, 2013; Munoz Descalzo *et al*, 2013).

At the phenotypic level, this situation is analogous to what is observed in homeostasis, where cell subpopulations are maintained in a dynamic equilibrium and therefore can be described as a system of homeostatic heterogeneities. The connection between the ES system and adult stem cells is emphasized by the observation that the signals that regulate the dynamics and structure of ES cell populations are FGF, Wnt and BMP which often appear as regulators of adult stem cell populations (Turner *et al*, 2014b).

A similar organization into dynamically balanced populations has been described in a compartment of the haematopoietic system, the erythroid/myeloid progenitor, characterized by the expression of the stem cell antigen 1, Sca1 (Pina *et al*, 2012). Although initially it was thought that this was a heterogeneous population in dynamic equilibrium (Huang *et al*, 2007; Chang *et al*, 2008), subdivision with additional markers, in particular the haematopoietic progenitor cell antigen CD34, reveals the existence of disparate subpopulations (Fig2D) (Pina *et al*, 2012). Cells with high CD34 are capable of self-renewal regardless of their levels of Sca1. A different subpopulation, with low Sca1 and CD34 levels, is analogous to the low Nanog one in ES cells, cannot reconstitute the culture and is committed to erythroid differentiation. Remarkably, the transcriptional state of this population of committed cells is still closer to the self-renewing cells (both Sca1 high- and low-level populations) than to cells differentiated to erythroids. Analysis of the networks underlying this balance indicates that the drift into these compartments is, *in vitro*, as is the case of the ES cells, stochastic, but once the cells have crossed this threshold, they are committed to differentiation (Chang *et al*, 2008; Pina *et al*, 2012; Teles *et al*, 2013). In the case of the ES cells, this population can be observed by cross-referencing the expression of Nanog with that of additional and independent pluripotency markers such as SSEA1 or PECAM (Fig2C; Canham *et al*, 2010; Lim, 2011).

Thus, within the TS framework, one can construe the CD34 or Nanog high state as the *o* state from which cells undergo a transition state and from there they either reverse or progress towards commitment [Sca1 low/CD34 low or low Nanog/PECAM(SSEA1) low], which involves a subpopulation irreversibly committed to differentiation (Fig2C and D). These observations, though still limited in number, raise the possibility that the heterogeneous molecular signature of stem cell populations reflects noisy gene expression regulated under a dynamic genetic programme, which can sustain a steady fraction of differentiating cells. Thus, although the fate of a self-renewing cell might be unforeseeable a priori, after the commitment, its behaviour is stereotyped. Moreover, the average dynamics of ensembles of self-renewing cells is also predictable, as are the ratios of differentiation cells. Thus, controlled heterogeneities in gene expression provide a basis to explain the stochastic lineages of stem cell populations.

## Similarities and differences between homeostatic and developmental systems: the example of the vertebrate retina

Homeostatic systems can be considered to be in a state of dynamic equilibrium, and the models that explain their behaviour account for this. However, the construction of a tissue or an organ is different from its maintenance. During organ development, fates are allocated to specific cell populations, sometimes in reproducible proportions and under specific genetic programmes that yield functional tissues. In some cases, the development of a tissue is associated with the activity of progenitors and stem cells that, in this case, display transient dynamics. A good example is the emergence of the vertebrate central nervous system from a population of progenitor cells that over time produces an array of neurons that create the sensory and motor systems (Wolpert *et al*, 2015). The number of final neurons is much bigger than the number of initial progenitors, and therefore, the progenitor population needs to be amplified. This has been studied in detail in the cortex (Qian *et al*, 2000; Shen *et al*, 2006) and, recently, the spinal cord (Kicheva *et al*, 2014). As the cell subpopulations at play (progenitors and DD) are similar to those operating in homeostatic systems, the question arises of how many of the principles applying to the dynamics of these populations also apply to developmental systems (see Box 2) and, specifically, whether stochastic processes apply. If the latter were the case, a reasonable question is how can the large variability introduced by such processes, which one might expect to be exponentially amplified by the net growth of the system, achieve a very stereotyped and reproducible final target. This issue has been explored in the vertebrate retina (He *et al*, 2012), and the results highlight some similarities but also some important differences between homeostatic and developmental systems.

### Cell growth and differentiation dynamics during retina development

**Figure 3:**
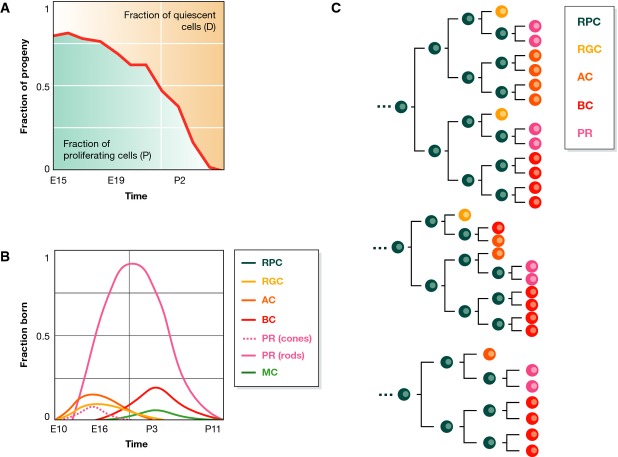
Vertebrate retinogenesis (A) During the development of the vertebrate retina, there is an initial phase where most of the divisions are symmetric proliferative leading to progenitor amplification. As development proceeds, proliferation slows down. When individual cells stop dividing, they differentiate and this leads to a link between the different cell types and the growth of the tissue (Livesey & Cepko, 2001). (B) Throughout retinal development, a reproducible sequence of overlapping temporal windows of specific fate adoption by differentiating cells is established. An early differentiating cell can become a retinal ganglion cell (RGC), a horizontal cell (HC), a rod photoreceptor (PR) or an amacrine cell (AC), whereas if it differentiates later, it can become a bipolar cell (BC), a Müller cell (MC) or a cone PR; that is, there appears to be an overlap between these windows of opportunities (adapted from Cepko *et al*, 1996). (C) Recent accurate single-cell tracing assays have unveiled complex lineage compositions in the zebrafish retina development. Three out of 60 clones from He *et al* (2012) are shown. Despite the observed high variability of clone compositions, there are some remarkable trends; for example, RGCs appear through asymmetric divisions, PRs through symmetric ones.

The retina is a structure with millions of cells structurally diversified into seven main functionally distinct cell types that are allocated in specific proportions and positions to generate a functioning organ (Masland & Raviola, 2000; Masland, 2001). The structure emerges over time during embryogenesis and continues growth after birth from a collection of retinal progenitor cells (RPG, P) which display two essential behaviours (Fig3A, Livesey & Cepko, 2001; Centanin *et al*, 2011; Centanin & Wittbrodt, 2014). The first one relates to the patterns of divisions which early on amplify the P compartment (P→PP, Fig3A and B) while towards the end of development lean towards terminally differentiating symmetric divisions (P→DD) (Livesey & Cepko, 2001; Rapaport *et al*, 2004) and in between exhibit a mixture of symmetric and asymmetric (P→PD) divisions, with the corresponding proportions varying over time. The second behaviour is a reproducible sequence of fates adopted by differentiating cells; although to date there is no way to reliably predict the fate of a specific cell when it divides, it is known that, at a given time, a cell always chooses among a restricted number of fates and that the repertoire of available fates changes with time (Cepko *et al*, 1996; Livesey & Cepko, 2001, Fig3B and C). These observations led to a model suggesting that as development proceeds, an intrinsically defined competence to obtain particular fates changes and this is what determines the fate of a differentiating cell. According to this model, a pattern of differentiating (DD) divisions is superimposed upon this shifting window of competence resulting in a loose but reproducible sequence of fates (Livesey & Cepko, 2001).

**Figure 4:**
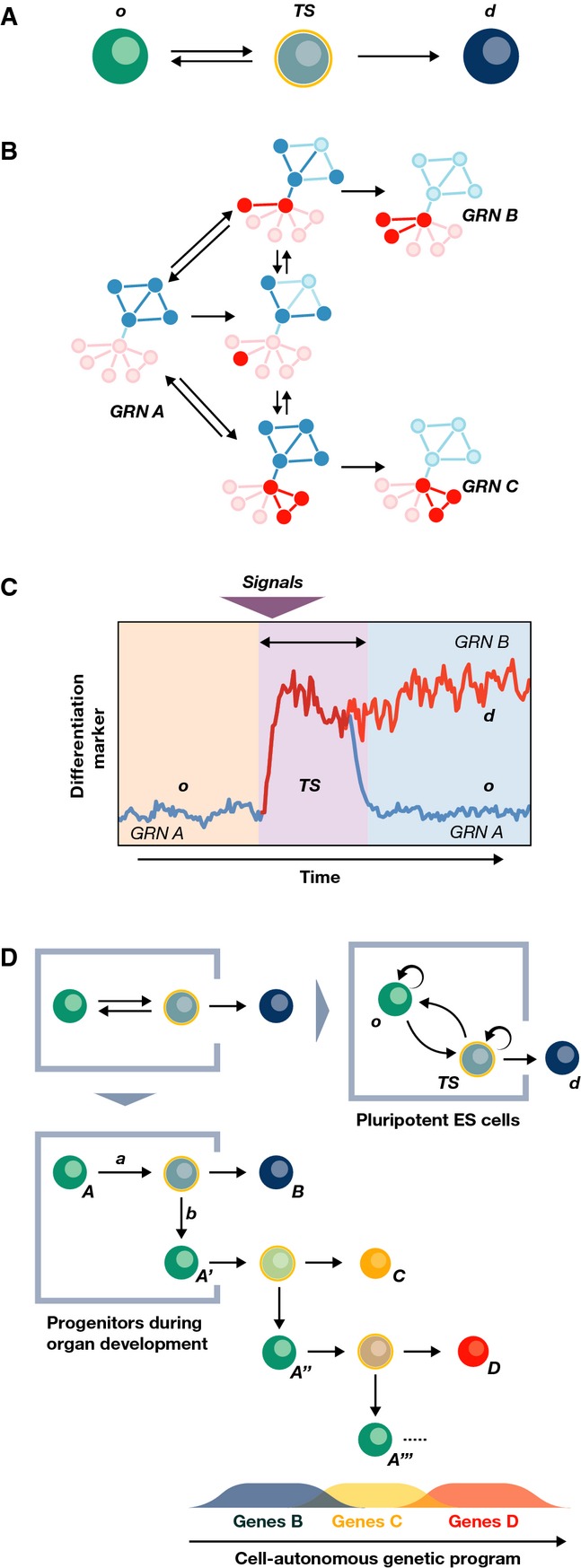
Molecular mechanisms of mediating fate decisions in development at the level of single cells (A) The transition state (TS, see Fig3) represents the basic unit for fate decisions. (B) During a fate transition, each cell executes a change of connectivity of their gene regulatory network from A to B. In this process, the cell will sample over time different configurations (microstates) of the available gene regulatory networks (GRN); many of these networks will resemble A, and therefore, the cell might have a chance to revert to the state of origin. When the network associated with fate B is connected, the cell moves to fate B. Within the TS, we suggest that cells are more susceptible to respond to signals that can bias their transcriptional state by affecting the connectivity (see text for details). (C) The TS state is an inherently noisy state, dominated by stochastic gene expression and affected by complex combinations of signals. As a result, the commitment or reversion event can be deemed as unpredictable at the level of individual cells. (D) The paradigm of the TS can be applied to pluripotent embryonic stem cells as well as to each differentiation step within the development of a tissue or organ. In the latter case, the TS is also controlled by a cell-autonomous genetic programme that establishes the order of appearance of the cellular fates, and thus, if a cell reverts to the state of origin, this might have changed in nature; this might account for many of the observations during retina development.

This classical model is similar to those suggested for the development of the cortex (Qian *et al*, 2000; Shen *et al*, 2006) and has been given quantitative substance through a detailed analysis of several lineages in the frog and rat retina in culture and the zebrafish retina *in vivo* (Wong & Rapaport, 2009; Gomes *et al*, 2011; He *et al*, 2012). These studies have provided support for an intrinsic mechanism of the fate assignment, the difficulty to assign specific fates to specific lineages and the need to balance over time the PP, PD and DD divisions in order to get the organ within a defined size. However, the studies in rat and fish (Gomes *et al*, 2011; He *et al*, 2012) have suggested that stochasticity, rather than a regulated programme, is the main driver in the fate assignment and tissue growth (Boije *et al*, 2014). While it is clear that it is not possible to predict the fate of a specific cell at a given time and that there is no simple pattern in the reported lineages, there is ample evidence for a reproducible sequence of fate assignment and for a restriction of the fates available to a cell at any given time: mixing or transplanting progenitors from different ages highlights the time restricted fate choice of the cells (Cepko *et al*, 1996; Belliveau & Cepko, 1999; Belliveau *et al*, 2000; Rapaport *et al*, 2001; Wong & Rapaport, 2009). Further support for a temporal programme of fate assignment is provided by the association of the expression of Ikaros with early and not late fates and by the complex, but numerable, sequence of expression of transcription factors (Mu *et al*, 2005; Wang & Harris, 2005; Ohsawa & Kageyama, 2008; Trimarchi *et al*, 2008), which makes it possible to predict lineage fate caused by the loss of a gene (Ohsawa & Kageyama, 2008; Andreazzoli, 2009). Regarding stochasticity, there is evidence that fate decisions are associated with heterogeneous gene expression (Trimarchi *et al*, 2008), but these might reflect priming for specific fates or transition states that cells pass through when they make decisions, rather than an open and unrestricted fate choice at any given time. In fact, in the context of a developing tissue, the TS could be crucial in determining how a population is subdivided, since the cells that return to ‘o’ would have the opportunity to adopt a new fate (Fig4D). Thus, in the retina, if the TS is short, a cell might not have time to make a decision, would remain in ‘o’ and would have to wait to another entry in the TS for a new fate to be desired. Depending on the programme of gene expression that is running, the choice the next time will be the same or different. This possibility provides an explanation for the perceived stochasticity of the system: it is during this transition state of finite time that the appropriate genes must be expressed in order to make the choice. The entries and exits of the TS would happen independently and simultaneously in many cells, and therefore, given the dimensionality of the system (with multiple genes and programmes of gene expression involved), a low sampling could give an impression of stochasticity.

In a recent review, Boije *et al* (2014) emphasize stochasticity, in the sense that all fates are available to all cells at any one time. We stress, however, that even though each individual cell might stochastically differentiate into one of the limited fates available, there are strong signs of tight regulation of the differentiation process, with most cell sub-types involved in the control of their temporal restriction. Thus, the complexity of this differentiation process might largely account for the reported lack of predictability of the fates adopted by individual cells, which the authors ascribe to stochasticity (see Fig3C).

## A molecular framework underlying fate decisions in developmental systems

The vertebrate retina shares a number of features with other developmental systems that need to be integrated and explained at the molecular level: (1) an asymptotic tendency towards a defined and reproducible size; (2) a temporal sequence of fate assignments; and (3) a certain degree of linkage between fate decision-making and the cell cycle. While stochastic models of fate assignment with no explicit regulation might account for the dynamic equilibrium of some homeostatic populations (Klein & Simons, 2011), it is not clear that they can be applied without modification to developmental systems (He *et al*, 2012). An important reason for this, which could also apply to homeostasis, is the need for coordinated control of the mode of cell division and fate adoption (Lander *et al*, 2009 and Box 2).

So far, there are a few lessons that can be learned from the analysis of the favoured competence model for the retina (Livesey & Cepko, 2001) and others that have been suggested for haematopoiesis (Pina *et al*, 2012) and ES cells (Trott *et al*, 2012). Here, we summarize these in the form of a set of premises that should be considered in any model for developmental systems:

- There exist ‘programmes’ of gene expression within single cells. Such ‘programmes’ are not a linear sequence of expression patterns but, rather, a network of gene interactions encoding combinatorial regulation through the differential activity and interactions of the network components over time.
- At each transition point of the programme, there are reversible intermediate transition states (TSs), where cells explore the space of available transcriptional networks (Fig4A and B),
- In the TS, individual cells make a choice for irreversible fate commitment at defined points of the programme (Fig4C).
- Signal-mediated cell interactions affect the progression to and from the TS and the choices made by individual cells (Fig4C).

## Conclusions: the transition state as the central element for fate decisions at the single cell level

With these premises in mind, we envision the TS as the central element of cell fate decisions at the single cell level (Martinez Arias & Hayward, 2006; Muñoz-Descalzo *et al*, 2012). This situation is similar to that described above for populations of stem cells, but in this case, the ‘*o*’ state is a particular intermediate in a developmental programme, say *A*, and the ‘*d*’ state is a new state, *B*, that is, the process is of the type *A→B* (notice that if *A→A or B,* we have a stem/progenitor cell population Fig4D). From this, it follows that underlying each cell fate decision, there exists a TS promoted by the transcriptional programme that leads from A to B and thus enables the fate choice. In the TS, a cell needs to dismantle the network associated with the A state and connect the corresponding B network. This process will require the connectivity of several nodes only a few of which will lead to a stable state B, and therefore, a cell will exit the TS when the B network is in place (Fig4A and B). The nodes of the networks that define these states are transcription factors and signalling effectors. While the transcription factors that define A initiate the transition by activating the B network, signalling might act by affecting the connectivity of the system in individual cells which will have an effect on the rates of state conversion (*o/A→TS, TS→o/A, TS→d/B*). As most signals are secreted, this will allow for a coordination of gene regulation at the population determining the number of cells that undergo a fate transition. In certain cases, some nodes might also include cell cycle-related proteins that, as recently shown in human ES cells (Pauklin & Vallier, 2013), can participate in cell fate choice, thus creating an opportunity to link the fate decision to the cell cycle (Rue *et al*, 2014).

The model that we propose to explain fate assignment from progenitor populations during development can be extrapolated to homeostatic systems on the premise that these are trapped TSs (Muñoz-Descalzo *et al*, 2012 and Fig4). The model also provides a framework to link transcriptional events with lineages by suggesting that the parameters of the TS, the rates of the gene regulatory networks at the transition state, determine the behaviour of a cell population. This notion suggests that the transition state could be an effective target for the balance of cell populations in development, homeostasis and, more important, pathological situations in which imbalances emerge between proliferation and differentiation.
